# Remarks on Tumor of the Sterno-Mastoid Muscle

**Published:** 1876-11

**Authors:** A. Reeves Jackson

**Affiliations:** Surgeon-in-Chief of the Woman’s Hospital of the State of Illinois; Lecturer on Diseases of Women, Rush Medical College, etc.


### EXAMINER.

Vol. XXXIII.—NOVEMBER, 1876.—No. 11.

### Original Communications.

### FURTHER REMARKS ON TUMOR OF THE STERNO-MASTOID MUSCLE.

By A. REEVES JACKSON, A.M., M.D.,

Surgeon-in-Chief of the Woman's Hospital of the State of Illinois; Lecturer on Diseases of Women, Rush Medical College, etc.

(Read before the Chicago Society of Physicians and Surgeons, June 12, 1876.)

At a meeting of this Society, held September 27th last, I presented the history of a case of tumor of the sterno-mastoid muscle of the right side, occurring in an infant soon after birth; and, at the same time, I called attention to a similar case which had been brought to the notice of the London Pathological Society by Dr. Frederick Taylor.*

Although several of the members of the London Society spoke as though they were more or less familiar with the disease mentioned by Dr. Taylor, I found that the literature of the subject was extremely meager; indeed, I could find no mention whatever of it in any systematic work to which I had access. Subsequently I found in the reprint of the London

* See this Journal, November, 1875, p. 820.

*Lancet* for 1863, p. 262, an account of a few cases, and I have thought that it might be of interest to bring them before the Society. Three of these cases were seen by Dr. Wilks, at the Royal Infirmary for Children, Waterloo Road:

CASE I. Was a female child seven weeks old. On examination a hard cord-like substance could be felt running in the direction of the sterno-mastoid muscle of the right side. Bending the head forward did not relax it, but caused it to be more readily grasped, and it was then found to be the muscle itself which was indurated. No enlarged glands were present. The child was healthy, and presented no appearances of syphilis. Mercury and chalk were administered, and an ointment of iodide of potassium was applied to the tumor. At the end of six weeks the hardness was fast disappearing.

CASE II. Male child, nine weeks old. The mother had noticed a hardness on the left side of the neck ever since birth. There was a hard substance corresponding in form and position to the sterno-mastoid muscle. It felt as if the muscle was changed into wood fiber. The child was healthy; there was neither syphilitic symptom or history.

It seems probable that, as the tumor was observed immediately after birth, it may have occurred in utero. The hardness disappeared under the use of iodide of potassium ointment, although Mr. Wilks thought that it might have done so spontaneously.

CASE III. The notes of this case were lost, but it was that of an infant a few weeks old, who had a similar hardness of the muscle of the right side, which was disappearing when last seen.

In the same volume I also found recorded two cases which came under the care of Mr. Paget, at St. Bartholomew's Hospital:

CASE I. A well-formed healthy child when two weeks old was observed to have two or three intensely hard lumps in the middle three-fourths of the left sterno-mastoid muscle. They felt like extreme induration of its substance, with enlargement. They were irregularly spheroidal, flattened and nodular. The muscle could not be fully lengthened, and the face could not be turned to the right side, although there was no wry-neck. It was thought that the disease was congenital.

The case was treated with iodine frictions and with small doses of iodide of potassium and cod liver oil. A year later the induration was scarcely perceptible, but the mastoid felt small and tough.

CASE II. In this case the child, likewise healthy, was two weeks old. A flattened lump about three-quarters of an inch in diameter involved the anterior part of the middle of the sterno-mastoid. The occasional application of iodine constituted the only treatment. In less than six months the swelling had almost disappeared.

Still another case is mentioned by Dr. Wm. J. Harris, in the *Lancet* for 1863, p. 421. The presentation at birth was a footling, which was afterwards found to be complicated by the funis twice encircling the neck. On the following day an unnatural hardness was felt on the right side of the child's neck, and a close examination showed that it was due to an induration involving the middle third of the sterno-mastoid muscle. The swelling was not noticeable when the face was directed straight forward, but when it was turned to the left side the hardened muscle could be plainly seen and was easily grasped.

A few days ago Dr. C. Paul Simon, a member of this Society, was kind enough to furnish me with a translation of a recent article by Blachez, in the *Gazette Hebdomadaire*, March 29, 1876, on a "Variety of Tumor of the Cervical Region in new-born Children," as follows:

"Last year, at the dispensary of l'Hôpital des Enfants, I had an opportunity of observing a peculiar kind of tumor of the neck, the nature of which at first sight seemed to me to be very obscure. Through a singular coincidence, two children affected with the same disease were presented to me at an interval of a few days, and within the same year we were able to collect a third observation. The particular conditions in which these children were found at the time of birth satisfied me very soon as regarded the pathogeny and nature of the tumors. I suggested to M. Planteau, my house surgeon, to make some researches on the subject, and to collect any analogous facts which might be found in the various French and foreign publications."

The results of M. Planteau's investigations are as follows:

"The three children which I have observed presented on the right side of the neck, in the thickness of the sterno-mastoid muscle, a tumor, hard, elastic, ovoid, non-fluctuating, producing very little pain, of the size of a pigeon's egg. This tumor had not been observed by the parents until two or three weeks after birth. Their attention was attracted by an inclination (sinking) of the head towards the right shoulder and rotation of the face towards the opposite side. The tumor was seated in the right sterno-mastoid muscle. The skin was of normal color, or a little roseate. When the chin was raised it was easier to form an idea of the tumor, which then resembled a strongly inflated spindle. On one of the children the muscle presented, besides, in the beginning of the disease, some small lumps on its middle portion. When the muscle was contracted the tumor was immovable; on the contrary, it was slightly movable when the muscle was relaxed. The muscle was entirely indurated in its middle part, but the induration of the lower half was most marked in the sternal fasciculus. The skin was not adherent to the tumor, and was not inflamed. There was no pain when the part was at rest, and the movements of the neck seemed to be but little interfered with. However, the child screamed when the tumor was pressed upon or the muscle contracted. These pains are most evident in the early days of the disease.

"It is difficult to completely straighten the head. Its sinking on the right side, and the rotation of the face towards the left, show that the sternal fasciculus is the part most affected. None of the children had syphilis. Two of them appeared perfectly healthy.

"We had no opportunity of making an anatomical examination of these tumors. We found in a case reported by Dr. Frederick Taylor* a notice on the composition of a tumor

*Loc. cit.

which seems to me to be analogous. The sternal extremity of the muscle was the portion principally indurated, and although presenting to the naked eye the characters of normal tissue, under the microscope the presence of a white fibrous tissue was manifest, its bundles separating the muscular fibers, which were intact. It was, apparently, a fibrous product, originating in the interstitial connective tissue, the result of an inflammation, the origin and growth of which we have now to study.

"Under the influence of contusions, frictions or tractions we know that a muscle may become the seat of an inflammatory process which, in a chronic stage, may result in fibrinous exudation.

"The circumstances under which these tumors are produced confirm these anatomical data. The three children were born in breech presentation. The extraction of the head lasted half an hour in the first; in the others long and energetic traction was used. The second was apparently dead when born.

"The tumor did not show in the first days of life, and was manifest only after a fortnight or three weeks. We could not learn anything about the position of the head, but it is well known that at the time of its expulsion the face is usually turned toward the sacrum.

"There seems to be an evident relation between the breech presentation, the violence used for the extraction of the head, and the nature of the lesion which we have described. This lesion is apparently the result of the twitching (jerking?) of the fibers of the sterno-mastoid muscle, determined by the traction exercised by the operator. Following this twitching, the muscle is the seat of an inflammation which results in the production of the fibrous interstitial tissue which forms the tumor. We say twitching, and not rupture of the fibers, because in the case in which an anatomical investigation was made the muscular fibers seemed to be intact. Besides, had a rupture existed, there would certainly have been an interstitial effusion of a certain quantity of blood, and the tumor would have been manifest a short time after birth. But, as we have already observed, its appearance was noticed only at a much more remote epoch, an interval necessary for the production of fibrous elements, the organization of the exudation and its growth.

"As a last proof, I may allude to the seat of the tumor, which, in our patients, was found always in the *right* sterno-mastoid muscle. If we consider the circumstances in which the head was extracted, the explanation is easily found. When the body is expelled, and the head remains in the pelvis with the face turned backwards, the right side of the child answers to the right hand of the operator placed in front of the woman; the operator supports the trunk of the child on his left hand and forearm, while with the right on its back he makes a few tractions. If the head does not come down, he introduces the right forefinger into the mouth of the child in order to move the chin. If this be done by a skillful operator, the twitching of the muscles on both sides will be equal; but if the operator be inexperienced, or over anxious to aid the delivery, he will endeavor to extract the head by means of energetic tractions rather than choose the most favorable position for the extraction. Without great care, it follows that traction will bear much more on the right side, and that the sterno-mastoid muscle of this side will be twitched and bruised. Besides, the neck being overstretched, the sternal bundle will be affected in particular."

The following are the conclusions at which the author arrives:

1. After the extraction of the child in pelvic presentations, one may see grow on the sterno-mastoid muscle tumors which seem to be of a fibrous nature.

2. These tumors are due to an interstitial myositis produced by exaggerated traction exerted on the sterno-mastoid muscle.

3. They are more frequent on the right side, apparently because of the habitual position of the fœtus in pelvic presentations.

4. These tumors do not bear any relation to syphilis.

5. They do not seem to have any grave character, although they are a long time in disappearing.

The foregoing cases, together with those formerly presented to the Society, and which have already been alluded to, are all that I have been able to find. With a single exception, in none of them was there any good reason to believe that a syphilitic taint had been inherited from the parents. Mr. Tatum, however, in an article on Affections of the Muscular System, in the third volume of Holmes' "System of Surgery," although he makes no allusion to the disease in children, states that the sterno-mastoid muscle is frequently affected in adults from syphilis, and he gives several cases in illustration.

Finally, in the *Gazette Hebdom.*, March 26, 1876, a case of tumor of the sterno-mastoid of the left side is reported by Dr. Pérate, in which the presentation was the first position of the head, and the labor terminated by forceps.

